# Deep Eutectic Solvents Application in Food Analysis

**DOI:** 10.3390/molecules26226846

**Published:** 2021-11-13

**Authors:** Cecilia Ortega-Zamora, Javier González-Sálamo, Javier Hernández-Borges

**Affiliations:** 1 Departamento de Química, Unidad Departamental de Química Analítica, Facultad de Ciencias, Universidad de La Laguna (ULL), Avda. Astrofísico Fco. Sánchez, s/n., 38206 San Cristóbal de La Laguna, Spain; cortegaz@ull.edu.es; 2 Instituto Universitario de Enfermedades Tropicales y Salud Pública de Canarias, Universidad de La Laguna (ULL), Avda. Astrofísico Fco. Sánchez, s/n., 38206 San Cristóbal de La Laguna, Spain; 3 Department of Chemistry, Sapienza University of Rome, P. le Aldo Moro 5, 00185 Rome, Italy

**Keywords:** deep eutectic solvents, green sample preparation, food analysis, solvent-based extraction techniques

## Abstract

Current trends in Analytical Chemistry are focused on the development of more sustainable and environmentally friendly procedures. However, and despite technological advances at the instrumental level having played a very important role in the greenness of the new methods, there is still work to be done regarding the sample preparation stage. In this sense, the implementation of new materials and solvents has been a great step towards the development of “greener” analytical methodologies. In particular, the application of deep eutectic solvents (DESs) has aroused great interest in recent years in this regard, as a consequence of their excellent physicochemical properties, general low toxicity, and high biodegradability if they are compared with classical organic solvents. Furthermore, the inclusion of DESs based on natural products (natural DESs, NADESs) has led to a notable increase in the popularity of this new generation of solvents in extraction techniques. This review article focuses on providing an overview of the applications and limitations of DESs in solvent-based extraction techniques for food analysis, paying especial attention to their hydrophobic or hydrophilic nature, which is one of the main factors affecting the extraction procedure, becoming even more important when such complex matrices are studied.

## 1. Introduction

Nowadays, humans are living in a globalized world, where it is necessary to guarantee the supply of food to a population of around 7900 million people, as well as to ensure its safe consumption, which becomes a difficult and essential task. The fact that each country has its own food regulations clearly complicates this scenario. In this sense, the development of new analytical methodologies that allow the effective and reliable analysis of foods, including the determination of pathogenic microorganisms and contaminants that can cause food poisoning or trigger food-related illnesses, are essential to guarantee the safety and quality of the food consumed around the world.

Current trends at both industrial and academia levels, are focused on the development and application of sustainable processes from an economical and environmental point of view. In this context, the development of new analytical processes is marked by the principles of the Green Analytical Chemistry (GAC), which emerged from the Green Chemistry principles in the 1990s, looking for a balance between an improvement in the quality of the results and the creation of more sustainable analytical procedures [1,2]. There is no doubt that the rapid development of analytical instrumentation, both in the miniaturization of the systems and in the improvement of sensitivity and selectivity, has contributed enormously in this regard. However, sample preparation still plays a very important role in any analytical process, especially in the determination of compounds at trace levels and/or very complex samples analysis, where the numerous interferences and the poor distribution of the analytes in the sample matrix make an enrichment of the target analytes and a clean-up of the sample necessary [1,3].

Different strategies have been followed in order to contribute to the development of new analytical procedures from a sustainable perspective. In this regard, many efforts have been made in the miniaturization of conventional sample preparation and separation techniques, as well as in the search for new materials and solvents that, after being used, have a low impact in the environment. In particular, since green chemistry was introduced, the search for alternatives to volatile toxic organic solvents has been one of the main challenges in sample preparation [4]. In this sense, several solvents of lower toxicity and improved properties (high thermal and chemical stability, adjustable viscosity, and good extraction capacity) have been introduced in this field, among which ionic liquids (ILs), switchable polarity solvents, supramolecular solvents and deep eutectic solvents (DESs) [4,5] can be found. Although all these new classes of solvents are playing a very important role in the development of new analytical processes, in recent years, DESs have aroused great interest due to their excellent physical–chemical properties and their eco-friendliness, which has led them to monopolize a large part of the latest publications related to solvent-based analytical techniques [6].

DESs, firstly introduced in 2003 by Abbot et al. [7], result from the combination of a hydrogen bond donor (HBD) and a hydrogen bond acceptor (HBA) at a specific molar ratio and temperature. This new generation of solvents is characterized by a lower melting point with respect to the HBA and HBD separately as a consequence of a charge delocalization produced by hydrogen bonds formation. Despite DESs have several features in common with ILs (easy synthetic procedures and variable viscosity, density and polarity), their synthesis is even cheaper and simpler, and they generally present lower toxicity, which have contributed to their popularity as extraction solvents [4,5].

The use of DESs in sample preparation have brought several benefits not only from an operational point of view, but also for the nontoxic and biodegradable nature of their constituents in many cases, such as quaternary ammonium and phosphonium salts, amines, alcohols, or carboxylic acids. However, the use of some non-environmentally friendly reagents during their synthesis is also quite common. As a result, many DESs still pose an environmental challenge because of their toxicity for living organisms [8]. In this sense, the latest trends have been focused on the preparation of natural DESs (NADESs), based on the use of natural products, such as amino acids, terpenes, sugars and natural organic acids, giving place to less toxic DESs with higher biodegradability and/or without toxicity, which undoubtedly contribute to the development of even more sustainable analytical procedures [4,5].

Since the unusual solvent properties of DESs at room temperature were shown, these have been classified into four categories attending to their components as a result of the wide variety of anionic and/or cationic species with which they could be formed [9]. Type I DESs are composed of non-hydrated metal halides and quaternary ammonium or imidazolium salts, while type II use hydrated metal halides as HBA. Type III DESs have shown particular versatility and have attracted the most attention, with applications in a wide variety of fields. This group includes DESs formed by mixing a quaternary ammonium salt (i.e., choline chloride, ChCl) with a wide range of HBDs that contain functional groups such as amides, carboxylic acids and alcohols. Considering that, the first DES designed by Abbott et al. [7], composed of ChCl and urea in a 1:2 molar ratio, and which was hydrophilic, could be classified as type III, as well as the DESs synthesized in the vast majority of the works collected in this review work. Finally, type IV is composed of a non-hydrated metal halide and a HBD.

As mentioned above, due to the great success of DESs, the number of publications related to their application in sample preparation techniques has grown rapidly, leading in some cases to the publication of incomplete and/or unreliable information. One of the issues that is often controversial is the definition of a DES, as the term “deep” should be clearly defined, since it is usual to find eutectic mixtures with the same starting components, but at different molar ratios. As an example, for the hydrophilic mixture between ChCl and oxalic acid, eutectic point properties have been described at molar ratios of 1:1 [10], 1:2 [11] and 1:3 [12]. However, it is not clear if all these combinations may be named as DESs, which highlights the great debate that exists on whether these mixtures could really be classified as DESs or, on the contrary, should be simply designated as “eutectic mixtures” or “eutectic melts”. There is also some debate associated with the hydrophobicity/hydrophilicity of a DES. Generally, it is stated that a water insoluble non-polar component as HBD and a water-soluble quaternary ammonium salt as HBA are necessary to obtain a hydrophobic DES (HDES). However, there is great controversy in this regard, since these types of solvents partially dissolve in water, leading to the loss of the DES. Thus, some authors have suggested that a DES should be defined as hydrophobic when all its components are insoluble in water, in such a way that they present sufficient stability in this type of solvent. Otherwise, they should be defined as “quasi-hydrophobic” DESs. In this sense, it is also worth mentioning the importance of carrying out characterization studies of the DES before and after the extraction procedure, since in many cases, especially when aqueous samples are analysed, the DES nature is finally lost, since water can act as HBA or HBD, resulting in a structure and/or polarity change [6].

Apart from the previously described, and despite the broad spectrum of DESs that can be synthesised, few information about the methodology to follow for the choice of the components of a DES to be used for specific applications can be found in the literature. Generally, hydrophilic DESs are suitable for the extraction of analytes from low-polar food samples, such as edible oils [13], fish [14] or rice flour [15], while HDESs are adequate for the extraction of inorganic and organic compounds from aqueous food samples, such as fruit juices [16], coffee [17] or tea beverages [18]. However, in certain cases, they can also be used in other matrices if a suitable dispersing or emulsifying agent is added to facilitate the analytes extraction, so this rule is not always fulfilled. Besides, there are many aspects affecting the extraction efficiency of the DES that make a prediction of the most suitable DES components, such as the polarity or acidity of the target analytes, as well as the viscosity of the resulting DES, which can be also affected by the addition of certain amounts of water.

This review article aims at providing a general overview on the application of DESs and NADESs as solvents in different solvent-based sample preparation approaches in food analysis. Considering the existing debate regarding the hydrophobicity of a DES, special attention has been paid to the hydrophilic or hydrophobic nature of the DESs applied in this field, due to the key role it plays during the extraction procedure, describing and discussing some specific and relevant applications.

## 2. Application of Hydrophilic Deep Eutectic Solvents

Since the first DES was synthesized in 2003 by Abbott et al. [7] and until 2015, most of the DESs reported in the literature were generally made up of hydrophilic compounds and, as a consequence, were soluble in water. Despite that fact clearly limits their application in certain sample preparation approaches, this type of DESs is still very useful for the extraction of different compounds of interest through simple, green and efficient procedures.

Among the main properties of hydrophilic DESs, their density values greater than that of water stand out [19]. Furthermore, and as mentioned before, these kinds of DESs are also characterized by their miscibility with polar solvents, such as water or methanol (MeOH), which is due to the hydrophilic nature of their components that contain highly electronegative groups and can form hydrogen bonds through special cases of dipole–dipole interactions [20]. Table 1 shows works in which hydrophilic DESs have been used for the extraction and determination of a great variety of analytes in different food samples. Most of the DESs shown in the table were prepared by simple mixing of their components with constant stirring and heating at temperatures below 100 °C until a transparent and homogeneous mixture was obtained, and in neither case a purification process was needed.

As can be seen in the table, hydrophilic DESs have been used with very good performances for the preconcentration and extraction of an extensive diversity of analytes, including both organic compounds (i.e., antioxidants [25], polycyclic aromatic hydrocarbons (PAHs) [65], pesticides [53,54,55,56], amino acids [24], phenolic compounds and caffeine [10,35,38,52,60], flavonoids [29,32], anthocyanins [31,34,58], mycotoxins [33,37], aflatoxins [39], sex hormones [36], antibiotics [41], preservatives [67], organophosphorus pesticides (OPPs) [44,57], curcumin [22,25,40,45], polybrominated diphenyl ethers (PBDEs) [43], and organochlorine pesticides (OCPs) [43,72]) and metals (Cd, Zn, As, Sb, Fe, Cu, Se, Mn, Pb, Cr, Co, Hg and Al [11,12,13,14,15,23,26,27,28,42,46,47,48,49,50,51,61,62,63,64,68,71]) from matrices of different natures and low polarity, such as edible oils [13,23,35,39,43,66,70,73], non-alcoholic beverages and fruit juices [38,44,53,54,56,57,62,67,71], vegetables [21,26,28,29,34,46,50,51,53,55,56,61,68], fruits [29,31,55,58], teas [22,40,42,49,50,65], spices [22,29,40], milk [24,41,48,62,63], water [12,46,62,63], fish tissues [11,14,27,42,46,47], eggs [62], flours [15,32,33,37,69], rice [64] and meat [42,46,51].

Taking into consideration the GAC principles, the downscaling of sample treatment has shown some advantages, such as low consumption of samples (10 μL–25 mL), low amounts/volumes of reagents and organic solvents (10–400 μL), a reduction and simplification of procedures, and high enrichment factors. For this reason, although some applications in which larger volumes of DES (even reaching 20 mL) can be found [21,24,29,32,35,37,41,58,69], most hydrophilic DESs have been applied in miniaturized liquid-based extraction techniques (see Table 1). In this sense, DESs have been applied in the three main modes of liquid-phase microextraction (LPME): dispersive liquid–liquid microextraction (DLLME), hollow-fibre liquid-phase microextraction (HF-LPME) and single drop microextraction (SDME). Among them, the speed, simplicity and low-cost of DLLME has made it the most widely used, allowing the preconcentration of different analytes in a wide variety of food matrices. It is important to highlight that in most cases, the hydrophilic DESs have been dispersed through various physical processes (manual agitation [53], ultrasound [14,30,38,39,44,46,47,50,51,61,62,63,70,73] or vortex stirring [12,13,22,33,40,43,45,48,49,52,59,63,69], temperature change [57], or air bubbled when pulling–pushing a syringe [42,55,64,71]), using few microliters of the extraction solvent and without the need for organic solvents. Additionally, some applications in which the drop obtained after the extraction stage has been solidified can be found [48,68], which allows the recovery of the complete drop and makes the procedure simpler, safer and faster [73].

However, and as it has been previously mentioned, the miscibility of hydrophilic DESs with water generally limits their direct application to aqueous samples. For this reason, in these cases, a dispersing or emulsifying agent (generally tetrahydrofuran, THF, or acetonitrile, ACN) is usually added to obtain a cloudy solution, achieving the separation of the two liquid phases after a shaking and centrifugation step in DES-based DLLME procedures [12,40,42,44,45,46,47,48,49,50,51,52,54,56]. Nonetheless, the addition of this solvent has negative consequences since it may reduce the environmental friendliness and increases the laboratory hazards. On the other hand, its properties, such as viscosity or density, are those that will allow better or worse retention of the analytes [1,6]. In general, hydrophilic DESs tend to have a relatively high viscosity, which makes an effective mass transfer in the extraction processes difficult, so a common practice to reduce their viscosity to suitable values consists in the addition of a known amount of water to hydrophilic DESs using the heating method [15,21,24,25,29,31,33,35,37], in which known concentrations of the three components (HBD, HBA and water) are mixed under constant stirring in a water bath (generally at 50 °C) until a homogeneous and transparent liquid is obtained. In certain cases, water has even been replaced by an organic solvent, such as ethanol (EtOH) or MeOH [43]. 

**Figure 1:**
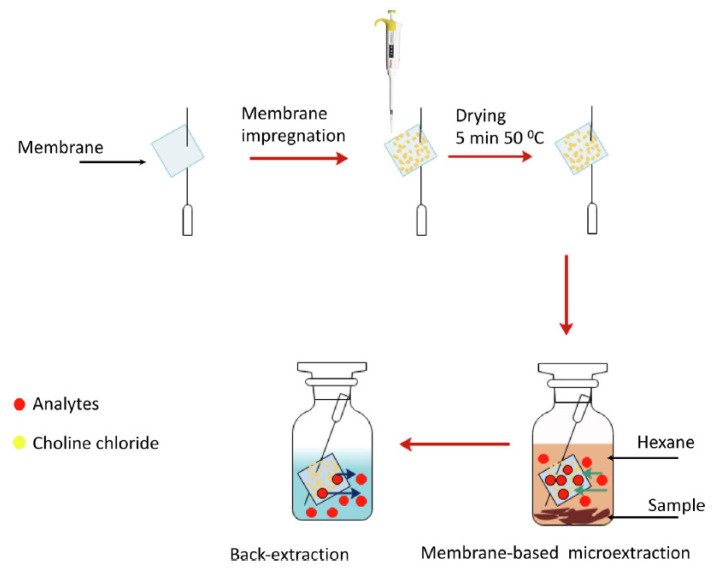
Deep eutectic mixture membrane-based microextraction process diagram. Reprinted from Shishov et al. [60] with permission of Elsevier.

Both viscosity and density values vary depending on the components of DES. For example, in the case of viscosity, DESs containing ChCl as HBA are more viscous when HBD is an acid (values up to 14,480 cP in ChCl:citric acid, 1:1 [74]) than when it is an alcohol (values lower than 400 cP [75]) due to the greater presence of hydrogen bonds between HBA and HBD. In addition, within the acids, citric acid contributes to a higher viscosity DESs, with values up to 437,768 cP (glucose:citric acid 1:1 [76]). Furthermore, the effect of the addition of water can be clearly observed in ChCl:citric acid (1:1) DES, whose viscosity decreases to 4080.8 cP [76] when amounts less than 50% of water are added, otherwise the eutectic properties would be lost, and dissolution of the individual DES components in water would occur. On the other hand, the change in the molar ratio also affects the viscosity of DES. In this way, in some cases if the proportion of ChCl is decreased, the viscosity is increased, such as ChCl:glycerol, which increases from 234 cP (1:1) to 301 cP (1:2), or ChCl:fructose, where it increases from 28.31 cP (1:1) to 72.42 cP (1:2) [75]. In the case of density, it can be ranged between 1.0 and 1.5 g/mL. The different values will depend on the molecular weight of the components, so for example, ChCl:maltose (1:2) will have a higher density (1.431 g/mL [77]) than ChCl:1,2-propanediol (1:2, 1.04 g/mL [78]), as if changing the HBA by one of higher molecular weight such as citric acid (citric acid:glucose, 1:1, 1.442 g/mL [76] compared to ChCl:glucose (1:1, 1.27 g/mL [79]). Furthermore, the addition of water decreases the density of DESs, for example, when adding 40% water to citric acid:glucose DES, the density decreases to a value of 1.246 g/mL [76]. From a procedural point of view, it is worth mentioning the work of Shishov and co-workers [60], in which the authors synthesized in situ different deep eutectic mixtures (DEMs) based on the combination of the analytes (phenols, HBDs) and ChCl (HBA) supported in a hydrophilic porous membrane. For this, square membranes (10 × 10 mm) were picked by syringe needle, as can be seen in Figure 1, and impregnated with a ChCl solution. After drying in an incubator, it was placed in a vial containing the sample mixed with hexane and shaken. After that, the syringe needle with the membrane was withdrawn, the hexane was evaporated and was introduced into a vial containing ultra-pure water. Then, it was shaken to promote analytes desorption, since this membrane type allows microextraction from organic sample phase and back-extraction of the analytes into aqueous phase. Finally, the aqueous phenol solution obtained was injected in the high-performance liquid chromatography (HPLC) system coupled to a fluorescence detector (FD). Several types of membrane were studied, being the poly(vinylidene fluoride-co-tetrafluoroethylene) the one that provided maximum extraction recovery. This membrane-based microextraction was used for the separation of six phenols from smoked sausages and smoked fish samples, showing good selectivity due to the formation of DEMs between ChCl and analytes. Good sensitivity with limits of detection (LODs) between 0.3 and 1.0 μg/kg and high extraction capacity with extraction recovery values ranging from 70 to 80% were obtained.

Hydrophilic DESs have also been combined with other materials and used in the extraction of the compounds of interest. For example, a magnetic nanofluid (MNF) consisting of a DES (ChCl:thiacetamide, 1:2 molar ratio) based magnetic multi-walled carbon nanotubes (MWCNTs) was successfully applied by Shirani et al. [71], combining the excellent properties of DESs with the magnetic features of magnetic nanomaterials. In this work, the authors sonicated the mixture of magnetic MWCNTs and the previously synthesized DES to obtain a homogenous black gel called DES-MNF. Then, the sample and the DES-MNF were mixed by rapidly suctioning and dispensing repeatedly for six times with a syringe, after which a turbid solution was obtained. The upper phase was eliminated by retaining the DES-MNF with an external magnet and a 1 M nitric acid solution was added to desorb the analytes. Finally, 10 μL of the supernatant solution were injected in an electrothermal atomic absorption spectroscopy (ETAAS) system. The proposed method showed high extraction capacity and good sensitivity for the determination of Cd, Pb, Cu and As from walnut, rice, tomato paste, spinach, orange juice, black tea and water samples.

In addition to the previously mentioned combinations, similar to what is made with ILs, DESs-based polymeric sorbents can also be synthesized and have been applied in food analysis. These poly(DES)s have emerged as promising alternatives to conventional sorbents used in solid-phase extraction (SPE) techniques, as they combine the properties of DESs and those of porous materials. As an example, Abdolhosseini et al. [68] prepared a DES of tetrabutylammonium bromide (TBABr) and acrylic acid (1:2 molar ratio) and polymerized it under solventless condition, through a cost-efficient and energy-saving photopolymerization process. DES polymerization consisted of mixing under a nitrogen atmosphere and at room temperature for 60 min the previously prepared DES, ethylene glycol dimethacrylate (used as crosslinker) and 2-hydroxy-4′-(2-hydroxyethoxy)-2-methylpropiophenone (used as photoinitiator) in a 100:10:1 weight ratio. The resulting homogeneous mixture was exposed to UV light and washed to remove any unreacted monomers. The synthesized polymeric DES was used for preconcentration of lead from vegetables such as onion, celery, carrot and tomato, as well as from mineral water samples through a dispersive SPE procedure, to later proceed to its quantification by flame atomic absorption spectroscopy. The results showed that the polymeric DES allowed obtaining acceptable selectively, low LOD (2 μg/L) and high stability since it can be reused 16 times without a significant reduction in the recovery.

As it can be seen, Table 1 also compiles several works in which hydrophilic NADESs have been used for the extraction of a wide variety of analytes. In order to demonstrate that their application constitutes a greener and more environmentally friendly alternative extraction procedure, in some of these works, its greenness has been evaluated according to the penalty points of an analytical eco-scale [80] calculated by considering hazards, amount of reagents, energy and waste, just like López et al. [24] did, who only obtained two penalty points in their developed method for the extraction of three free seleno-amino acids in lyophilized samples of seleno-biofortified sheep milk and cow milk powder and determination by liquid chromatography (LC)-inductively coupled plasma-mass spectrometry (ICP-MS). Another method used to evaluate the toxicity of hydrophilic NADESs has been bacterial growth inhibition. In this way, Huang et al. [30] performed a culture with two Gram-positive (*S. aureus* and *L. monocytogenes*) and two Gram-negative (*E. coli* and *S. enteritidis*) bacteria, which were incubated in a nutrient agar medium with a filter paper soaked with each of the thirteen NADESs they tested to extract rutin from tartary buckwheat hull. It was shown that none of the NADESs led to a decrease in the growth of bacteria with the exception of glycerol:L-arginine NADES, because, despite the fact that the individual components are nontoxic and were approved by the European Food Safety Authority [81,82], there occurs a charge delocalization as a result of a hydrogen bond, which makes the eutectic mixture toxic [83].

After the application of the DESs as extraction solvents in the above-mentioned procedures, analytes have generally been determined by HPLC or ultra-high-performance liquid chromatography (UHPLC) using different detection systems, such as UV [21,29,30,31,32,44,52,57,70,73], diode array detector (DAD) [10,25,35,36,59,67], FD [37,39,60,65], tandem mass spectrometry (MS/MS) [33], as a result of their appropriate solubility in the mobile phase, or ICP-MS, this last for free seleno-amino acids determination [24]. However, they have also been separated and detected by UV-Vis spectrophotometry [14,22,38,40,45], atomic absorption spectroscopy (AAS) [11,23,47,68], slotted quartz tube-flame AAS [48,49], hydride generation AAS [12], graphite furnace AAS [15,26,42], ETAAS [13,27,46,50,51,71] and ICP-optical emission spectroscopy (OES) [28]. Gas chromatography (GC) coupled to electron capture detection (ECD) [54,72] or flame ionization detection (FID) [53,55,56] have also been used, although in a very reduced number of applications. Furthermore, in some cases, after performing the developed method, samples were injected into a GC-MS for better identification of the analytes. However, in other cases, as previously commented, because the DESs used are highly viscous, they had to be mixed before performing the extraction technique with a solvent, such as MeOH or EtOH, to decrease their viscosity and, in this way, avoid irreproducibility problems during injection into the chromatographic system. As an example, the work of Solaesa and co-workers can be highlighted [43], who synthesized a DES based on ChCl and phenol with a 1:2 molar ratio, which they mixed in a 1:1 (*v*/*v*) ratio with EtOH to be used in the extraction of five PBDEs and three OCPs from fish oils using vortex-assisted liquid–liquid microextraction (VA-LLME)-GC-MS/MS with 5′-fluoro-3,3′,4,4′,5-pentabromodiphenyl ether and triphenylphosphate as internal standards.

The great extraction capacity shown by the hydrophilic DESs together with the sophisticated detection techniques used, have allowed to obtain low LODs, in the order of μg/L or μg/kg in most cases as can be seen in Table 1, or even ng/L or ng/kg [12,13,39,42,46,65,71].

## 3. Applications of Hydrophobic Deep Eutectic Solvents

Despite the previously mentioned limitations of hydrophilic DESs, it was not until 2015 that van Osch et al. [84] presented some DESs with hydrophobic properties for the first time. These DESs were characterized by the immiscibility of their two components with water, resulting in a low water content after being mixed with this solvent (approx. 1.8 wt%) and a low leaching of the quaternary ammonium salts (approx. 1.9 wt%). These hydrophobic solvents consisted of a long chain alkyl quaternary ammonium salt (e.g., tetrabutylammonium chloride (N_4444_Cl), methyltrioctylammonium chloride (N_8881_Cl), tetraheptylammonium chloride (N_7777_Cl), tetraoctylammonium chloride (N_8888_Cl), methyltrioctylammonium bromide (N_8881_Br) and tetraoctylammonium bromide (N_8888_Br)) and poorly soluble carboxylic acids (e.g., decanoic acid), and their extraction capacity was evaluated by extracting volatile fatty acids from diluted aqueous solutions. Since then, multiple HDESs based on neutral compounds have also been proposed, including combinations of monoterpenes with fatty acids [85], tetraalkylammonium halides with fatty acids and alcohols [86,87], fatty acids with fatty acids [88], and monoterpenes with monoterpenes [17]. Many of the HDESs have also been designed and classified following the same classification that had been previously proposed and that was already used for hydrophilic DESs (type I, II, III and IV), but due to their need to be stable in the aquatic environment, they are mainly grouped into type III (a combination of a quaternary salt (HBA) with a HBD) and type IV (a combination of metal chloride with HBD) [89].

The main difference between hydrophilic and hydrophobic DESs lies in the presence of long alkyl chains or cycloalkyl groups, which reduces the effect of hydrophilic zones (e.g., charges of the salts) and hydrophilic groups (e.g., carboxylate and hydroxyl groups) [1,90]. These eutectic mixtures have unique properties of density, acidity, polarity, viscosity and volatility, which provide a good extraction capability through a careful selection of their components [89]. As a consequence, it has been found that the extraction efficiency of HDESs depends to a great extent on their immiscibility with water as a function of the difference in density. Thus, in contrast to hydrophilic DESs, HDESs generally have lower density values than water [19], since the increase in the length of the alkyl chain of the salt components results in a decrease in density (within 0.80–1.10 g/mL) [91], although, for example, DESs containing fluorinated alcohol (e.g., hexafluoroisopropanol, HFIP) are generally denser than water (around 1.5 g/mL) [59]. In addition, it is necessary to take into account that the greater the difference in density between DES and water, the more easily the separation between the two phases will occur [91].

On the other hand, most HDESs have a melting temperature below 25 °C, which allows them to be used as solvents or reaction media in different applications at room temperature [90,92]. However, it should be noted that increasing the alkyl chain of the component acting as HBD, like carboxylic acids, increases the melting point of HDESs, while increasing the alkyl chain of the ammonium salt results in a lower melting point [90]. It is also important to highlight that the hydrophobicity of DESs is also affected by the structure of their individual components in such a way that the longer the alkyl chain of the components (both in HBA and HBD), the lower the solubility in the aqueous phase of each of them as well as of the DES [89]. Regarding the viscosity of HDESs, it is usually high because the hydrogen bonds that are established between its components, decrease the movement of the HDES molecules. However, these eutectic mixtures, like the hydrophilic ones, show a wide range of viscosity (between 2.6 and 5985.0 cP), since it depends on the components that make up the HDES, especially the one that acts as HBA, which allows us to design solvents for specific tasks depending on their handling capacity [90]. Among them, it is possible to differentiate between neutral HDESs (such as menthol:decanoic acid 1:2, 27.7 cP) that are less viscous than ionic HDESs (such as N_4444_Cl:decanoic acid 1:2, 265.3 cP), and within the latter, the HDESs that contain the bromide anion (such as N_8881_Br:decanoic acid 1:2, 576.5 cP) are more viscous than those that contain the chlorine anion (such as N_8881_Cl:decanoic acid 1:2, 472.6 cP) [91]. Likewise, as in hydrophilic DESs, an increase in temperature leads to a decrease in viscosity [93,94].

As mentioned above, HDESs are mainly characterized by their immiscibility in the aqueous phase. For this reason, it is necessary to study the stability of HDESs in contact with water, so that there is no leaching or loss of its components towards the aqueous phase, as well as that their water content is practically zero [84]. Many of the HDESs formed through the combination of a hydrophobic and a hydrophilic component have been found to be unstable in water. This is because the hydrophilic component tends to leach into the aqueous phase. For example, Florindo et al. [95] showed that the DES formed by DL-menthol:dodecanoic acid (2:1, molar ratio) was stable in contact with water compared to DL-menthol:acetic acid (1:1, molar ratio) and N_4444_Cl:octanoic acid (1:2, molar ratio) when comparing the 1H NMR spectra of each one of them. As a consequence, Shishov et al. [6] proposed a new term for these unstable HDESs in aqueous phase: “quasi-hydrophobic DES”, since it would not be appropriate to consider them as HDESs. In most of the works collected in this review, a study has not been carried out to verify if the extraction is due to a HDES or to one of its components as consequence of the leaching of the other one, as it was verified, for example, in the study carried out by Ortega-Zamora et al. [96]. That is why in this section both the HDESs and the quasi-hydrophobic DESs that have been used for the analysis of food samples, have been grouped and are shown in Table 2.

In general, all the DESs included in the table have been synthesized following the same guidelines as for hydrophilic DESs: mixing the components while heating with constant stirring until a homogeneous mixture is obtained. In addition, although most HDESs used in food analysis are made up of two components, some ternary DESs have been designed, which show numerous advantages over traditional DESs, such as a lower viscosity and melting point, and even a better extraction efficiency in some cases [17,114]. It is the case of the work of Shishov and co-workers [103], in which different quaternary ammonium salts, carboxylic acids and medium chain fatty acids were studied as components of a DES. The best results were obtained with the DES formed by TBABr:malonic acid:hexanoic acid (1:1:1 molar ratio) and it was used in the sequential extraction of sulfonamides from chicken samples through a DLLME followed by HPLC-UV. First, an attempt was made to carry out the extraction using a DES formed by TBABr and hexanoic acid but, due to its high viscosity and lack of acidic media, a high mass-transfer from the solid phase did not occur. However, with the introduction of a third component, in this case a carboxylic acid, an increment in the extraction efficiency was observed due to the formation of hydrogen bonds between hexanoic acid and the analytes. The introduction of hexanoic acid not only has benefits during the extraction process, but also produces a decrease of DES viscosity, enabling its direct injection in the chromatographic system. Some HDESs have even been mixed with other materials, such as Fe_3_O_4_ magnetic nanoparticles (m-NPs) to form a nanoferrofluid, which speeds up the sample preparation procedure and makes it more sustainable to perform the preconcentration of various analytes in complex food samples before their injection in different chromatographic systems [17,120]. For its preparation, the Fe_3_O_4_ m-NPs and the DES are separately synthesized and then mixed under constant stirring until a homogeneous fluid is obtained: the DES-based nanofluid [17,120]. As an example, Fan et al. [17] synthesized one of these nanofluids based on a ternary HDES composed of menthol, borneol and camphor in a 5:1:4 molar ratio. They used it for the extraction of 14 PAHs in 12 kinds of coffee samples after four different roasting conditions, which were separated and determined by HPLC-FD. LODs in the order of ng/L for all analytes and recovery values between 91.3 and 121%, showed the excellent performance of the methodology, which allowed verifying that the content of PAHs in the samples of coffee was modified depending on the temperature and time conditions of the roasting of its beans.

Currently, studies on the synthesis and application of HDESs for the extraction of a great variety of analytes from food matrices have expanded rapidly, which has led to an increase in the number of articles published in recent years. As can be seen in Table 2, several HDESs have been used for the extraction of both organic (phthalic acid esters [18,96,99], dyes [97,104,110,117], PAHs [17,85], sterols [98], pesticides [100,105,112,122,124,126,127], herbicides [102], insecticides [125], preservatives [101], pigments [86], antibiotics [87,103,108,114], fluorescent whitening agents [106], vitamins [107], mycotoxins [16], bisphenols [115], perfluoroalkyl substances [120] and terpenes [121]) and inorganic compounds (Co, Cd, Ni, As, V and Pb [109,111,113,116,118,119,123]) from aqueous phases (water [96,113,118,124], soft drinks [18,85,86,96,110], infusions [18,86,102,124], coffee [17], dairy products [86,99,108,111,114,126], fruit juices [16,86,87] and wine [116]). However, sauces [97,104], oils [97,100,120], egg yolk [97,125], jelly [110,117], honey [105] and solid food (meat [103], fish [106], flours [107], spices [121], dried fruits [112,119], vegetables [98,113,118,122,123] and fruits [101,115,118]) samples have also been analysed using HDESs. It is important to mention that, due to the complexity of some of the studied matrixes, different previous treatments have been needed in most cases. In this way, water [96,113,118,124], infusions [18,86,124], and soft drinks [18,85,86,96,110] were analysed without additional treatment or after degasification or filtration, while others such as honey [105] or fruit juices [16,86] were analysed after dilution with water and, in some cases, filtration. Likewise, procedures such as lyophilization or a previous extraction with an organic solvent (e.g., n-hexane, ACN, MeOH or acetone) were very useful in the treatment of egg yolk, olive oil, chili sauce, honey and some fruit juices, although it may be contradictory with the development of green sample preparation procedures using DESs. As a specific example, a hydrophilic DES has even been used in the treatment of oil samples to reduce the matrix effect [100]. In those cases, in which the matrix is more complex, more laborious procedures previous to the extraction of the compounds of interest are needed. For example, in the case of milk or yogurt, a deproteinization is usually carried out with ACN [114], although (NH_4_)_2_SO_4_ [108] has also been employed. When it comes to solid samples such as dried fruits, cereals, onion, parsley or even dairy products (milk, yogurt, cheese, etc.), they are usually digested with H_2_O_2_:HNO_3_ (1:3, *v*/*v*) [109], although in some cases only HNO_3_ is used [119,123], before the application of microextraction techniques. However, if a previous step such as QuEChERS (quick, easy, cheap, effective, rugged and safe) [112], is carried out before the extraction technique in dried fruits for example, it would only be necessary to grind and homogenize them.

**Figure 2:**
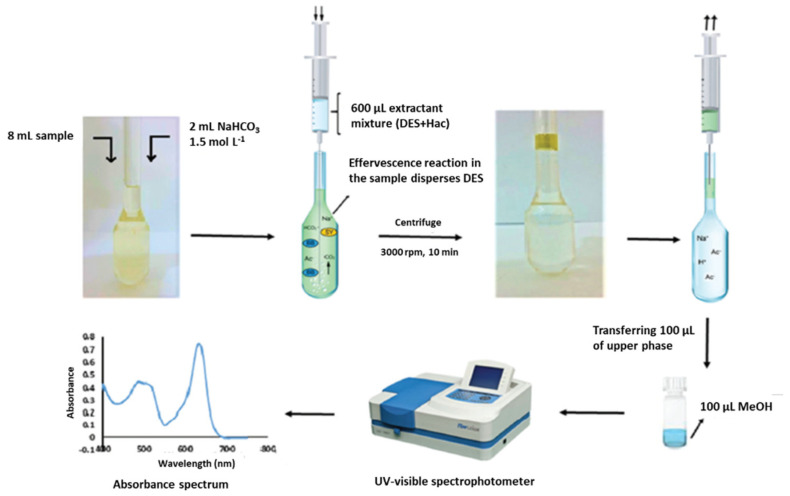
Schematic diagram of effervescence assisted dispersive liquid–liquid microextraction. Reprinted from Ravandi and Fat’hi [117] with permission of Royal Society of Chemistry.

Nowadays, the above-mentioned low solubility of HDESs in aqueous samples has allowed their large application as extraction solvents in microextraction methods, complying with the principles of GAC. Among the different variants of the LPME, DLLME constitutes once more one of the preferred options for the application of HDES for the analysis of samples of diverse nature, including water [96], soft drinks [85,110], infusions [124], honey [105], dried fruits [119], flour [107], fruit juices [124] and egg yolk [125]. In this sense, it is important to highlight that in certain cases, no additional solvents have been necessary to obtain a good dispersion of the HDES into the sample [18,86,96,97,98,110]. Instead, the DLLME procedure has been assisted in different ways, including the vortex-assisted DLLME [86,97,99,100,104,106], ultrasound-assisted DLLME [16,85,87,101,113,116,123,124], air-assisted [109] and microwave-assisted DLLME [128]. However, other versions have also been used, such as DLLME based on the solidification of the floating organic drop (SFO) [18,115,122,126]; salting out-DLLME-back extraction or salt induced-homogenous liquid-liquid extraction-DLLME, in which a salt is added (e.g., NaCl, (NH_4_)_2_SO_4_, Na_2_SO_4_ or NH_4_Cl) to reduce the solubility of the analytes in water and to increase their distribution coefficients in the organic phase [108,114]; effervescence-assisted DLLME in which an effervescent reaction between a proton donor solvent (acetic acid, which has been previously mixed with HDES in a 3:1 (*v*/*v*) ratio) and an effervescent agent (sodium bicarbonate) is produced generating carbon dioxide that facilitates the dispersion of the extraction solvent (HDES, see Figure 2) [117]; or even the combination of DLLME with a previous extraction and clean-up stage, such as the QuEChERS method, in which the extracted supernatant was used as a dispersant in the following DLLME for further purification and preconcentration [112]. Another interesting modification of the LPME technique is the use of the above-mentioned ferrofluid as an extraction solvent [17,120].

On the other hand, in order to improve the sensitivity towards volatile compounds, headspace microextraction techniques have been used, such as headspace SDME, which is faster and cheaper than other extraction methods, and allows extracting a wide range of components with diverse physicochemical properties in matrices where no pre-treatment is necessary. As an example, Triaux et al. [121] used a HDES (tetrabutylammonium bromide (N_4444_Br):dodecanol, 1:2 molar ratio) as extracting solvent for the extraction of 67 terpenes from six spices (cumin, cinnamon, clove, fennel, nutmeg and thyme) used as bought without additional grinding. The DES was introduced into the needle of a GC microsyringe, which was inserted into the headspace of the vial where the sample was located. The DES was pushed to form a drop at the tip of the needle and was left for 90 min at 80 °C for the absorption of the volatile analytes on the DES drop. The extracts were analysed by GC-MS obtaining limits of quantification (LOQs) between 0.47 and 86.40 μg/g.

Regarding the final determination of the analytes, different separation and detection techniques have been applied, being LC, both HPLC [17,18,86,87,96,97,98,99,101,103,104,107,110,112,114,124,125] and UHPLC [115,120], the most extensively used, although some applications of micellar electrokinetic capillary chromatography [108] and GC [85,100,102,105,121,122,126,127] can also be found mainly for non-ionic HDESs, which are characterized by a higher volatility. These separation techniques have been coupled to different detection systems, including UV [18,87,96,97,99,103,107,110,112,124,125], DAD [86,104,114], variable wavelength detectors [98], FD [17,101], MS [102,120,121,122], MS/MS [85,115], FID [105,126,127] and ECD [100]. Other techniques have also been directly applied without a previous separation of the analytes, such as UV-Vis spectroscopy [16,117], fluorescence spectrometry [106] and AAS [109,111,113,116,118,119,123]. The combination of these techniques with the outstanding extraction performances shown by the synthesized HDESs has provided excellent sensitivity in all cases, with LODs in the low ppb level.

As with hydrophilic DESs, hydrophobic NADESs can also be synthesized from natural compounds immiscible with water. As mentioned above, apart from all the inherent advantages of HDESs, they fully represent the GAC principles, since they are easily prepared, cost-effective and are not harmful to the environment [129]. In addition, as a result of their diverse compositions (among the components most used as HBA, menthol, thymol and camphor stand out, while carboxylic acids are usually used as HBDs), they have a wide range of polarity and physical properties [8]. Hydrophobic NADESs have also been used in food analysis, although they are still not very abundant compared to the use of “non-natural HDESs”, as shown in Table 2. It is worth highlighting the work of Soltani and co-workers [100], who synthesized a hydrophobic NADES composed of thymol and vanillin (1:1, molar ratio in which a transparent yellow liquid remained). They calculated its solubility in water (0.005%, *w*/*v*) and its octanol/water distribution constant (log K_OW_ = 4.30) to verify its hydrophobicity. Furthermore, they found that the thymol:vanillin (1:1) DES was stable for at least one week at ambient conditions. The authors used it as an extraction solvent in VA-LLME coupled with GC-μECD for the determination of 16 pesticides in olive oil samples (extra virgin, virgin and refined olive oils), complex food samples that showed a high matrix effect. Therefore, the authors developed a DES-based liquid–liquid solvent system (n-hexane/ACN/DES) to achieve cleaning the sample as much as possible and, thus, improve sensitivity and reduce the matrix effect. To do this, they mixed the sample with n-hexane, and then with ACN (used as extraction solvent) and a hydrophilic NADES composed of ChCl and urea. After shaking and centrifuging, a triphasic system was observed in which the medium layer was the ACN that contained the pesticide residues and was used for performing the preconcentration procedure worked up. The develop method provided high recovery percentages for the analytes (between 63.1–119.4%) with high precision (relative standard deviation values in the range 2–7%), and it was also simple and sensitive with LODs in the range 0.01–0.08 μg/kg.

## 4. Conclusions

Considering the current trends in the Analytical Chemistry field, DESs constitute a very interesting alternative to conventional solvents, not only because of their interesting physicochemical properties, but because they have made it possible to develop more sustainable analytical procedures, from an environmental point of view due to their low toxicity, and also from an economic point of view due to their general low cost and the simplicity of their synthesis. As with other solvents widely used in sample preparation techniques, the wide variety of HBD and HBA available make it possible to configure a large number of DESs, which has allowed their application for the extraction of a large number of organic and inorganic analytes from diverse food matrices. In this sense, the hydrophilic or hydrophobic nature of DES plays a fundamental role since it has a great influence on the extractive process. However, few studies have evaluated this aspect, as well as their toxicity. In fact, the greenness of many DESs currently used as green solvents has not been fully even evaluated, and many of them actually continue to present significant toxicity or pose a risk to the environment, in many cases due to synthesis procedures that use conventional solvents. In this context, the introduction of NADESs opens a window of hope for reducing the impact of this type of solvent on the environment.

It is also important to mention that, even with their previously mentioned great properties, there are still some aspects that limit the application of DESs to the analysis of food samples from an operational point of view, such as the miscibility of the hydrophilic ones with aqueous samples or the need of including organic solvents to provide a good dispersion of the DESs or to decrease their viscosity, which goes against the principles of developing sustainable analytical methodologies. Besides, the limited number of cheap, readily available and biodegradable components (especially in the case of hydrophobic NADESs) for the synthesis of HDESs also pose a limitation for their use in food analysis.

Despite the aforementioned problems that the use of DESs still poses today, this type of new solvents still has a wide margin for improvement and are proposed as an alternative for the future to be taken into account, not only at an analytical level, but also in a wide range of applications.

## Figures and Tables

**Table 1:** Application of hydrophilic DESs in sample preparation procedures for food analysis.

DES (Molar Ratio) (Volume)	Analytes	Sample	Sample Preparation	Extraction Technique	Separation and Detection Technique	LODs	Recovery % (RSD %)	Comments	Reference
**NADES**									
ChCl:oxalic acid (1:1) (-)	17 polyphenols	*Aegle marmelos* (Bael/woodapple) (500 mg)	Samples were frozen, freeze-dried and pulverized	UAE	HPLC-DAD	-	- (-)	DES was mixed with 25% of water (*v*/*v*).	[10]
ChCl:oxalic acid (1:2) (2500 μL)	Cu, Fe and Zn	Muscle, liver and gills fish tissues (100 mg)	Samples were freeze-dried, ground to fine powders and sieved through a 125 mesh	An acid digestion method with HNO_3_ 1 M	FAAS	6–53 μg/L	95–100% (-)	The reproducibility of the method was validated by analysing all samples in different laboratories by ICP-OES. For comparison, a CAD was used for the determination of analytes in all samples.	[11]
ChCl:oxalic acid (1:3) (700 μL)	As and Sb	Waste, mineral, well, tap, and river water, honey and rice (125 mL)	Water samples were filtered. Rice samples were dried, ground and homogenized	VA-DLLME	HG-AAS	0.0075–0.0156 μg/L	94–104% (1–3%)	7 DESs were evaluated. An extraction with MeOH:H_2_O (1:1, *v*/*v*) was done to ensure the reliability of analysis results. THF was used as an emulsifying agent.	[12]
ChCl:urea (1:2) (160 μL)	Pb and Cd	Sesame, soybean, olive, sunflower and corn oils (28,000 mg)	Samples were used without any sample pretreatment steps	VA-DLLME	ETAAS	0.0002–0.008 μg/kg	95–104% (-)	The quality assurance/quality control procedure was performed to ensure the obtained results.	[13]
BeHCl:sorbitol (1:3) (600 μL)	MeHg and total Hg	Fish (tuna, salmon, trout, mackerel, whiting and anchovy), seafood (shrimp) and lake, dam, well and waste water (2.5 mL)	Edible parts of fish were homogenised, oven-dried and frozen. Water samples were filtered and concentrated by evaporation	UA-DLLME	UV-Vis	0.25–0.92 μg/L	90–104% (2–5%)	Different pretreatment for each Hg species. NADES phase contained 10% water (*v*/*v*). ACN was used as aprotic solvent. NADES acts as a reactive pH-controlled zwitterionic surfactant.	[14]
ChCl:L-(+)-tartaric acid:water (1:1:2) (2000 μL)	Cd	Rice flour (300 mg)	Samples were dried	UAE	GFAAS	-	- (-)	20 NADESs were evaluated. The regeneration of Cd-contaminated NADESs was explored. After the UAE, a conventional acid microwave extraction was performed.	[15]
Lactic acid:levulinic acid (1:1) (1500 μL)	20-hydroxyecdysone	Spinach (100 mg)	Samples were dried and ground	VA-DLLME	UHPLC-UV	170 μg/kg	88–93% (3–9%)	DES contained 30% of water. The recovery of the analyte was higher with NADES-SLE than IL-SLE procedure.	[21]
BeHCl:glycerol (1:3) (500 μL)	Curcumin	Cinnamon tea, anti-parasite herbal tea, herbal tea, mixed herbal tea, tumeric, curry, cinnamon and sesame (1.5 mL)	Samples were ground, homogenized and extracted with MeOH	VA-DLLME	UV-Vis	1.5 μg/L	90–108% (2–4%)	8 alcohol-based DESs were prepared.	[22]
Glycolic acid:mandelic acid (2:1) (750 μL)	Cd(II) and Zn(II)	Fish oil, butter and margarine (7 mL)	Samples were diluted with ethyl acetate	RP-DLLME	FAAS	0.12–0.18 μg/L	89–104% (3–10%)	3% (*v*/*v*) HNO_3_ solution was used as extraction solvent.	[23]
Lactic acid:glucose (5:1) (3090 μL)	3 Se-amino acids	Milk (940 mg)	Samples were lyophilized and powdered	UAE	LC-ICP-MS	7.37–9.64 μg/kg	86–109% (<7%)	The DES was mixed with 18% water (*v*/*v*). The extraction with NADES has less penalty points of AES than other techniques.	[24]
Sucrose:lactic acid:water (1:5:7) for extract curcuminoids and fructose:lactic acid:water (1:5:5) for antioxidant extraction (-)	Curcuminoids and antioxidants	Turmeric (-)	Sample was ground	MAE	HPLC-DAD to quantify the curcumin	-	37–41% (-) (AR)	5 NADESs were evaluated and 4 of them showed better results than the ones obtained with MeOH:H_2_O (4:1, *v*/*v*). FCCD was used for optimization. CUPRAC method was used to determine the antioxidant capacity.	[25]
ChCl:oxalic acid (1:2) (2500 μL)	Se and As	Mushroom (100 mg)	Samples were dried at 105 °C for 24 h	An acid digestion method with HNO_3_ 1.5 M	GFAAS	0.32–0.50 μg/L	96–100% (-)	There are no significant differences between the extraction with DES and the conventional wet acid digestion method.	[26]
ChCl:oxalic acid (1:2) (2000 μL)	Se and As	Fish and canned fish (80 mg)	-	An acid digestion method with HNO_3_ 1 M	ETAAS	0.46–0.75 μg/kg	94–99% (-)	-	[27]
ChCl:tartaric acid ChCl:oxalic acid ChCl:citric acid (1:1) (1000 μL)	Mn	Basil herb, spinach, dill and cucumber barks (830 mg for tartaric and oxalic acid-based DESs and 1250 mg for citric acid-based DES)	Samples were dried, crushed and gridded to fine particles	Samples were extracted with DES for 2 h at 95 °C, centrifugated, filtrated and diluted	ICP-OES	0.34, 0.50 and 1.23 μg/L (oxalic, tartaric, citric acids, respectively)	82–114% (-)	All three DESs showed good results as extractants.	[28]
AcetylChCl:lactic acid (2:1) (600 μL)	8 flavonoids	Cranberry, fruits of *Lycium barbarum* L., grape, plum, orange peel, onion, broccoli, mustard, rosemary and black pepper (200 mg)	Samples were dried, milled and stored in paper bags at ambient temperature for 4–5 months	VA-DLLME	UHPLC-UV	150 μg/kg	70–94% (-)	Better recovery values were obtained when adding 30% (*v*/*v*) of water to the DES. Chrysin was used as IS. The extraction method was compared with an UAE method. Optimization was done with a CCD.	[29]
ChCl:glycerol (1:1) (1000 μL)	Rutin	Tartary buckwheat hull (40 mg)	The sample was ground to powder	UA-DLLME	HPLC-UV	-	- (-)	13 NADESs were studied. The toxicities of the NADESs were evaluated with two Gram-positive and two Gram-negative bacteria. Closed bottle test was used to determine the biodegradability of the NADESs.	[30]
ChCl:citric acid:glucose (1:1:1) (100000 μL)	Anthocyanins	Mulberry (5000 mg)	-	HSH-CBE	HPLC-UV	-	- (-)	PBD and BBD were carried out to determine optimum extraction conditions. HSH-CBE was compared with other extraction methods. DES was mixed with 30% (*v*/*v*) of water.	[31]
ChCl:citric acid (1:1) (600 μL)	4 isoflavones	Soybeans, flour, pasta, breakfast cereals, cutlets, tripe, soy drink, soy nuts, soy cubes and dietary supplements (200 mg)	Samples were grounded and dried. In the case of the dietary supplements, the contents of 10–20 capsules were pooled	UA-DLLME	UHPLC-UV	60–140 μg/kg	65–99% (-)	30% (*w*/*w*) water in NADES was used. CCD were used to determine the optimum conditions.	[32]
ChCl:urea (1:2) (1000 μL)	6 mycotoxins	Cricket flour, silkworm pupae and black cricket powder (150 mg)	Samples were homogenized	VA-DLLME	UHPLC-MS/MS	10–110 μg/kg	49–104% (1–13%)	FFD was used to determine the optimum conditions. DES was supplemented with 15% of Milli-Q water. 3-hydroxy-5-methylphenyl-2,4-dihydroxybenzoate was used as IS.	[33]
ChCl:citric acid (1:1) (-)	Anthocyanins	Black carrot (-)	-	UAE	-	-	- (-)	Five DESs were prepared. DESs were added to samples with a sample/DES ratio of 1:4. The biodegradability of the tested DESs were >80% after 28 days.	[34]
Glucose:lactic acid:water (1:3:3) (5000 μL)	Phenolic compounds	Extra virgin olive oil (1000 mg)	Purified olive oil was obtained after an omics approach	LLE	HPLC-DAD	-	- (-)	-	[35]
ChCl:urea (1:2) (40 µL)	3 sex hormones	Milk (20 mL)	Samples were mixed with TCA (a protein coagulant), centrifuged and the supernatant was diluted	VA-DLLME	HPLC-DAD	1.0–1.3 μg/L	80–116% (3–14%)	MMWCNTs were used as sorbent in mSPE. The DES with the analytes is adsorbed on the surface of MMWCNTs.	[36]
ChCl:urea (1:2) (20000 μL)	Ochratoxin A	Durum wheat, bread crumbs, biscuits and bran (4000 mg)	Samples were grounded	SLE	HPLC-FD	0.09 μg/kg	42–88% (2–11%)	The DES contained 40% (*w*/*w*) of water. Samples were purified/concentrated with IMA columns.	[37]
ChCl:urea (1:2) (565.1 μL)	Caffeine	Cola, energy drink, ice tea, instant coffee, espresso, dry coffee, chocolate and ice cream (2 mL)	Beverages were degassed, diluted, sonicated and filtered. Food samples were ground, sieved, sonicated with boiling water and filtered	UA-DLLME	UV-Vis	7.5 μg/L	93–107% (1–2%)	CCD was used to determine the optimum conditions.	[38]
ChCl:malonic acid (1:2) (30 μL)	4 aflatoxins	Corn, soybean, peanut and rapessed oils (5000 mg)	Samples were diluted with n-hexane (1:9, *v*/*v*)	UA-DLLME	HPLC-FD	0.0005–0.003 μg/kg	72–113% (1–9%)	-	[39]
ChCl:maltose (1:3) (762.5 μL)	Curcumin	Tea, honey and spices (5 mL)	Samples were mixed with water, sonicated and filtered	VA-DLLME	UV-Vis	0.1 μg/L	94–103% (1–3%)	THF was used as emulsifier solvent.	[40]
ChCl:glycerol (1:2) (2000 μL)	2 antibiotics	Milk (0.5 mL)	Sample was deproteinized with ACN (1% NH_3_)	DLLME	LC-MS/MS	-	83–87% (-)	The DES was mixed with chloroform (2:1, *v*/*v*). DES was also used to modify MIPs which were used as a sorbent in SPE. Recovery values in SPE were higher than in the DLLME procedure.	[41]
**Non NADES**									
ChCl:phenol (1:4) (600 μL)	Pb(II)	Black and green tea, cumin, cow and chicken meat, linseed, canned fish, potato, and lake, waste, river and sea water (30 mL)	Water samples were filtered and food samples were digested with microwave system	AA-DLLME	GFAAS	0.0006 μg/L	97–99% (2–3%)	THF was used as a demulsifying solvent. 4-(2-thiazolylazo) resorcinol (0.1%, *w*/*v*) was used as a complexing reagent.	[42]
ChCl:phenol (1:2) (180 μL)	5 PBDEs and 3 OCPs	Fish oil (300 mg)	-	VA-DLLME	GC-MS/MS	0.2–0.7 μg/kg	64–110% (0–7%)	FBDE-126 and TPP were used as ISs. The greenness of the procedure was assessed using the AES. DES was mixed with EtOH 1:1 (*v*/*v*) to improve reproducibility.	[43]
ChCl:phenol (1:2) (408 μL)	2 OPPs	Red grape and sour cherry juices (10 mL)	Samples were filtered	UA-DLLME	HPLC-UV	0.070–0.096 μg/L	87–117% (4–10%)	THF was used as an emulsifier agent.	[44]
ChCl:phenol (1:4) (400 μL)	Curcumin	Herbal tea, turmeric drug, turmeric powder and root herbal tea (10 mL)	Herbal tea samples were extracted with boiling demineralized water and HNO_3_ was used for stabilizing the solutions. Solid samples were powdered and extracted with MeOH	VA-DLLME	UV-vis	2.86 μg/L	96–102% (1–6%)	HPLC-DAD was used to check the accuracy of the developed method. Different molar ratios of DES composition were studied. THF was used as an emulsifier agent.	[45]
ChCl:phenol (1:4) (500 μL)	Cd	Bean stew, black tea, chicken shawarma, canned corn, corn, canned mushroom, cheese, mushroom, fish tissue, tomato, meat, canned fish, rice and spinach, drinking, tap, and waste water, and ice tea (50 mL)	Solid food samples were digested by a microwave system. Water samples were filtered	UA-DLLME	ETAAS	0.000023 μg/L	98–100% for liquid samples and 99% for reference materials used in solid samples (-)	Azo was used as a complexing agent for Cd. THF was used as an emulsifying agent. The optimization was assessed using a factorial design. The proposed technique was compared with other reported methods.	[46]
ChCl:phenol (1:2) (600 μL)	Zn	Fish and eel (10 mL)	Samples were digested with HNO_3_:H_2_O:H_2_O_2_ (1:3:2 mL ratio) and diluted	UA-DLLME	FAAS	0.041 μg/kg	93–101% (2–5%)	8-hydroxy quinoline was used as a chelating agent. THF was used as an emulsifier agent.	[47]
ChCl:phenol (1:2) (500 μL)	Pb	Milk (8 mL)	-	VA-DLLME	SQT-FAAS	8.7 μg/L	102–103% (1–6%)	Detection power was improved by 48 times using this method with respect to conventional FAAS system. THF was used as an emulsifying agent.	[48]
ChCl:phenol (1:2) (600 μL)	Co	Linden tea (10 mL)	Linden samples were boiled in water and filtered	VA-DLLME	SQT-FAAS	2.0 μg/L	97–100% (3–5%)	THF was used as an emulsifier agent.	[49]
ChCl:phenol (1:3) (1000 μL)	As(III) and As(V)	Edible mushrooms, sediment, green tea, black tea, rice, soil, cigarette, and lake, river, tap and mineral water (25 mL)	Water samples were filtered. Food and environmental samples were digested with HNO_3_ (65%, *w*/*w*)	UA-DLLME	ETAAS	0.01 μg/L	96–99% (3–4%) for water samples and 98% (-) for reference materials of mushroom and fish tissue	DDTC was used as a chelating agent. THF was used as dispersive solvent. As(V) was reduced to As(III), and total arsenic was determined.	[50]
ChCl:phenol (1:4) (500 μL)	Al(III)	Drinking, river, mineral, sea and spring water, rice, cultivated mushroom and chicken meat (25 mL)	Food samples were used after a microwave digestion	UA-DLLME	ETAAS	0.032 μg/L	97–100% (2–4%)	THF was used as extraction solvent. PBD was used to determine the optimum conditions.	[51]
ChCl:phenol (1:3) (400 μL)	Caffeine	Coffee (5 mL)	Coffee was grounded and mixed with water. The mixture was heated and centrifuged	VA-DLLME	HPLC-UV	120 μg/L	91–101% (-)	THF was used as emulsifier solvent.	[52]
ChCl:4-chlorophenol (2:1) (142 µL)	5 pesticides	Apple, grape and sour cherry juices, and fresh beer, cucumber, potato and tomato (5 mL)	All juices were diluted with water at a ratio 1:3. Vegetables were squeezed, centrifuged and the supernatants were diluted 1:5	DLLME	GC-FID	0.13–0.31 μg/L	86–99% (3–7%)	Diazinon was detected in the tomato samples. Temperature was a key factor in this method.	[53]
ChCl:4-chlorophenol (1:2) (145 μL)	7 pesticides	Apple, pineapple, cherry, peach, and red and green grape juices (10 mL)	Only peach juice was diluted with water (1:1, *v*/*v*)	DLLME	GC-ECD	0.006–0.038 μg/L	71–115% (-)	dSPME with mGO functionalized was used before DLLME step. ACN was used as disperser.	[54]
ChCl:4-chlorophenol (1:2) (190 μL)	9 pesticides	Apple, onion, cucumber, tomato and grape juice (5 mL)	Fruits were squeezed and diluted, while grape juice was used without dilution	GA-DLLME	GC-FID	0.24–1.4 μg/L	86–107% (-)	-	[55]
ChCl:4-chlorophenol (1:2) (132 μL)	6 pesticides	Grape, apple and orange juices, lettuce, carrot, onion, cucumber, tomato and garlic (5 mL)	Orange juice was centrifuged and filtered, and all fruit juices were diluted. Vegetables were crushed, centrifuged and supernatant was diluted	DLLME	GC-FID	0.46–3.1 μg/L	87–101% (4–7%)	ACN was used as a disperser solvent.	[56]
ChCl:4-chlorophenol (1:2.5) (200 μL)	2 OPPs	Fresh juice of apple, peach and orange, and tap and well water (6 mL)	Juice samples were centrifuged, filtered and diluted. Water samples were diluted	TC-DLLME	HPLC-UV	0.15–0.30 μg/L	96–105% (-)	This method can be applied in saline samples with an ionic strength up to 0.5 M.	[57]
ChCl:1,2-propanediol (1:2) (15000 μL)	7 anthocyanins	*Lycium ruthenicum* Murr. fruit (1000 mg)	Samples were dried, ground and sieved	UA-SLE	Off-line heart-cutting 2D HPLC-DAD/MS	36 μg/L	- (-)	DES contained 10% (*v*/*v*) water. Extraction optimization was done using BBD.	[58]
HFIP:L-carnitine (2:1) (150 mg)	5 pyrethroids	Black, green and oolong teas, and apple, red grape and purple grape juices (5 mL)	Samples were centrifuged and the supernatant was filtered	VA-DLLME	HPLC-DAD	0.06–0.17 μg/L	85–109% (1–8%)	ACN was used as dispersion solvent. L-carnitine-based DESs provided higher EF than betaine-based DESs.	[59]
ChCl:phenol (-) (-)	6 phenols	Smoked sausage and smoked fish (200 mg)	Samples were homogenized and store one month before use them	MME	HPLC-FD	0.3–1.0 μg/kg	70–80% (-)	GC-MS was used as a reference procedure. In situ DES formation between analytes (HBDs) and ChCl (HBA) supported in a hydrophilic porous membrane.	[60]
ChCl:phenol (1:2) (350 μL)	Cr(III) and Cr(VI)	Tap and river water, mushroom and soybean (10 mL)	Food samples were extracted with HCl and filtered	UA-DLLME	FAAS	0.4 μg/L	92–106% (2–4%)	PAN was used as a chelating agent. THF was used as an aprotic solvent. UA-LPME was superior than VA-LPME. Cr(VI) was reduced to Cr(III) with L-ascorbic.	[61]
ChCl:phenol (1:3) (500 μL)	Se(IV) and Se (VI)	Tap and mineral water, ice tea, cow milk, mixed fruit juice, orange juice, grape fruit, sheep milk, yogurt, honey, egg, canned fish and eddible mushroom (25 mL)	Water samples were filtered. Food samples were digested with H_2_O_2_ and HNO_3_	UA-DLLME	ETAAS	0.0046 μg/L	96–99% (1–4%)	THF was used as aprotic solvent. 3,3-DAB was used as complexing agent.	[62]
ChCl:phenol (1:3) (500 μL)	Se(IV) and Se(VI)	Infant formula milk, infant cereal, tap and mineral water (200 mg)	Milk and cereal samples were digested in a microwave with HNO_3_ and H_2_O_2_ (2:1, *v*/*v*). All samples were filtered	UA-DLLME and VA-DLLME	GFAAS	0.029 (UA-DLLME) and 0.036 (VA-DLLME) μg/L	98–99% (UA-DLLME) and 96–98% (VA-DLLME) (-)	Na_2_S_2_O_3_ was used to reduce Se(VI) to Se(IV). UA-DLLME required less time and showed better LOD, RSD and EF than VA-DLLME. THF was used as an aprotic solvent.	[63]
ChCl:phenyl-EtOH (1:2) (250 μL)	Cr(III) and Cr(VI)	Tap, river and mineral water, and rice and sausage (10 mL)	Food samples were digested with HCl and all samples were filtered	AA-DLLME	FAAS	0.4 μg/L	86–105% (1–2%)	PAN was used as a complexing agent.	[64]
ChCl:HFIP (1:2) (60 μL for solid samples and 160 μL for liquid samples)	6 PAHs	Milkvetch, ginseng, honeysuckle, Maojian tea and Anji white tea (500 mg). Jazmine tea beverage, natural mineral water, white grape and litchi juices, and honey (5 mL)	Solid samples were ground into powder and sieved, liquid samples were centrifugated and filtered, and honey was diluted	DT-DLLME	HPLC-FD	0.00005–0.0042 μg/L	88–114% (0–10%)	ACN was used as emulsifier and density regulator. Depending on the amount of emulsifier, the DES-rich phase could appear in the bottom or in the top phase. Rhodamine B was added as an indicator of the DES-rich phase.	[65]
ChCl:3,3-dimethyl butyric acid (1:1) (15 μL)	4 OPPs	Sunflower, sesame, olive, canola and corn oil (2.5 mL)	-	DLLME-SFO	GC-NPD	0.06–0.24 μg/L	84–100% (2–8%)	dSPE is used before DLLME-SFO for a better performance in edible oil samples easily. PSA was selected as the sorbent in dSPE.	[66]
TBABr:acetic acid (1:2) (200 μL)	6 preservatives	Functional, tea and carbonated drinks (4 mL)	-	DLLME-SFO	HPLC-DAD	20–50 μg/L	78–101% (0–4%)	1-decanol was used as extractant. NaCl was added. BBD was used to determine the optimum conditions.	[67]
TBABr:acrylic acid (1:2) (10 mg)	Pb(II)	Tap and mineral water, onion, celery, carrot and tomato (50 mL)	Vegetables were dried at 100 °C and digested with HNO_3_ and H_2_O_2_	dSPE	FAAS	2.0 μg/L	92–106% (1–5%)	DES was polymerized under solventless condition. Sorbent can be reused 16 times without significant decrease in the recovery. Results were compared with the obtained using ICP-MS.	[68]
ChCl:ethylene glycol (1:2) (3500 μL)	Gliadin	Heat-untreated (flour) and heat-treated (crackers and biscuits) gluten-free food (350 mg)	Food samples were milled to fine powder	VA-SLE	ELISA	-	78–113% (3–13%)	The extraction capacity of the DESs was compared with the one of the EtOH-water medium. ChCl:urea DES provided better results, but DES with ethylene glycol provided the best performance in terms of recovery.	[69]
ChCl:ethylene glycol (1:2) (50 μL)	Ferulic, caffeic and cinnamic acids	Olive, almond, sesame and cinnamon oils (2 mL)	Samples were diluted with n-hexane (1:1, *v*/*v*)	UA-DLLME	HPLC-UV	0.39–0.63 μg/L	95–105% (2–5%)	Extraction optimization was done using BBD.	[70]
ChCl:thiacetamide (1:2) (40 μL)	Cd, Pb, Cu and As	Walnut, rice, tomato paste, spinach, orange juice, black tea, and tap and river water (48 mL)	Black tea sample was mixed with HNO_3_ 1:1 and heated, and food samples were mixed with HNO_3_ (65%) and H_2_O_2_ (30%) and heated. All samples were filtered	AA-DLLME	ETAAS	0.003–0.0042 μg/L	94–101% (2–3%)	The extraction solvent was a magnetic nanofluid (a mixture of mCNTs and DES).	[71]
DEAC:pivalic acid (1:2) (80 μL)	4 OCPs	Cocoa powder and cocoa beans (1000 mg)	Cocoa bean samples were crushed	DLLME	GC-ECD	0.011–0.031 μg/kg	84–99% (3–8%)	ACN was used as an extraction solvent and as a dispersive solvent in DLLME.	[72]
Tetramethylammonium chloride:ethylene glycol (1:3) (30 μL)	3 plant growth regulators	Safflower, olive, camellia, colza and soybean oils (1 mL)	Samples were diluted with n-hexane (10% oil and 90% n-hexane)	UA-DLLME	HPLC-UV	1200–7500 μg/L	73–108% (0–9%)	-	[73]

2D: two-dimensional; 3,3-DAB: 3,3’-diaminobenzidine; AA: air-assisted; ACN: acetonitrile; AES: Analytical Eco-Scale; AR: absolute recovery; Azo: (Z)-N-(3,5-diphenyl-1H-pyrrol-2-yl)-3,5-diphenyl-2H-pyrrol-2-imine; BBD: Box-Behnken design; BeHCl: betaine hydrochloride; CAD: conventional acid digestion; CBE: cavitation-burst extraction; CCD: central composite design; CE: conventional extraction method; ChCl: choline chloride; CUPRAC: cupric reducing antioxidant capacity; DAD: diode-array detector; DDTC: sodium diethyldithiocarbamate; DEAC: N,N-diethanol ammonium chloride; DES: deep eutectic solvent; DHP-d_4_: dihexyl phthalate-3,4,5,6-d_4_; DLLME: dispersive liquid–liquid microextraction; DLPME: dispersive liquid-phase microextraction; dSPE: dispersive solid-phase extraction; dSPME: dispersive solid-phase microextraction; DT: density-tunable; ECD: electron capture detection; EDLLME: emulsification dispersive liquid–liquid microextraction; EF: enrichment factor; ELISA: enzyme-linked immunosorbent assay; ETAAS: electrothermal atomic absorption spectrometry; EtOH: ethanol; FAAS: flame atomic absorption spectrometry; FBDE-126: 5′-fluoro-3,3′,4,4′,5-pentabromodiphenyl ether; FCCD: face centered composite design; FD: fluorescence detector; FFD: fractional factorial design; FID: flame ionization detector; GA: gas assisted; GC: gas chromatography; GFAAS: graphite furnace atomic absorption spectrophotometer; HBA: hydrogen bond acceptor; HBD: hydrogen bond donor; HF: hollow fibre; HFIP: hexafluoroisopropanol; HG-AAS: hydride generation–atomic absorption spectrometry; HPLC: high-performance liquid chromatography; HSH: high-speed homogenization; ICP: inductively coupled plasma; IL: ionic liquid; IS: internal standard; LC: liquid chromatography; LLE: liquid–liquid extraction; LLME: liquid–liquid microextraction; LOD: limit of detection; LPME: liquid-phase microextraction; MAE: microwave assisted extraction; mCNT: magnetic carbon nanotube; ME: microextraction; MeOH: methanol; mGO: magnetic graphene oxide; MIP: molecular imprinted polymer; MME: membrane-based microextraction; MS/MS: tandem mass spectrometry; MS: mass spectrometry; mSPE: magnetic solid-phase extraction; MWCNT: multi-walled carbon nanotube; NADES: natural deep eutectic solvent; NPD: nitrogen phosphorus detector; OCP: organochloride pesticide; OES: optical emission spectrometry; OPP: organophosphorus pesticide; PAE: phthalic acid ester; PAH: polycyclic aromatic hydrocarbon; PAN: 1-(2-pyridylazo)-2-naphtol; PBD: Plackett–Burman design; PBDE: polybrominated diphenyl ether; PSA: primary secondary amine; RP: reversed-phase; RSD: relative standard deviation; RSM: response surface methodology; SFO: solidification of the floating organic drop; SLE: solid–liquid extraction; SPE: solid-phase extraction; SQT: slotted quartz tube; TBABr: tetrabutylammonium bromide; TC: temperature-controlled; TCA: trichloroacetic acid; THF: tetrahydrofuran; TPP: triphenylphosphate; UA: ultrasound-assisted; UAE: ultrasound assisted extraction; UHPLC: ultra-high-performance liquid chromatography; US: ultrasound; UV: ultraviolet; VA: vortex-assisted; Vis: visible.

**Table 2:** Application of hydrophobic or quasi-hydrophobic DESs in sample preparation procedures for food analysis.

DES (Molar Ratio) (Volume)	Analytes	Sample	Sample Preparation	Extraction Technique	Separation and Detection Technique	LODs	Recovery% (RSD%)	Comments	Reference
**NADES**									
Menthol:borneol:camphor (5:1:4) (80 mg)	14 PAHs	Coffee (8 mL)	Samples were roasted in four different conditions	Nanoferrofluid	HPLC-FD	0.00031–0.0059 μg/L	91–121% (1–11%)	NADES modified Fe_3_O_4_ mNPs presented excellent microextraction performance.	[17]
L-menthol:acetic acid (1:1) (100 µL)	9 PAEs	Green tea, tonic, lime and lemon drink (20 mL), and camomile, pennyroyal mint and linden teas (15 mL)	Infusions were prepared with hot Milli-Q water. All samples were previously degasified	DLLME-SFO	HPLC-UV	1.05–15.33 µg/L	71–125% (1–22%)	DHP and DNOP were used as ISs.	[18]
Camphor:hexanoic acid (1:1) (175 μL)	15 PAHs	Herbal ready-to-drink beverages (10 mL)	-	UA-DLLME	GC-MS/MS	0.01 μg/L	69–125% (1–17%)	CCD was applied to evaluate the main factors affecting the process. ACN was used as dispersive solvent.	[85]
L-menthol:acetic acid (1:1) (100 μL)	9 PAEs	Tap and mineral water and sparkling apple juice (20 mL)	Water samples were applied without any previous treatment, while the soft drink was degassed	DLLME	HPLC-UV	1.08–6.90 μg/L	71–120% (1–20%)	DHP and DNOP were used as ISs.	[96]
ChCl:sesamol (1:3) (800 μL)	Sudan I	Chili oil, chili sauce and duck egg yolk (200 mg)	Chili sauce and duck egg yolk were extracted with n-hexane	VA-DLLME	HPLC-UV	20 μg/kg	93–118% (-)	-	[97]
DL-menthol:pyruvic acid (1:2) (20000 μL)	Ergosterol	Mushroom (1000 mg)	Mushrooms were washed, shredded, lyophilized and pulverized without peeling off the skin	UA-SLE	HPLC-VWD	-	- (-)	39 HDESs were evaluated. DES can be reused for up to six extraction cycles. CCD was selected for optimization.	[98]
Menthol:lauric acid (1:1) (400 μL)	7 PAEs	Milk (5 mL)	Sample was centrifuged with ACN, MgSO_4_ and NaAc. The supernatant was also centrifuged in the same conditions. The final supernatant was diluted	VA-DLLME	HPLC-UV	1.06–4.55 μg/L	84–107% (2–4%)	NaOH and HCl were used as emulsifier and phase separation agent, respectively.	[99]
Thymol:vanillin (1:1) (200 μL)	16 pesticides	Olive oil (500 mg)	Samples were vortexed with n-hexane and extracted with ACN. Then, the hydrophilic ChCl:urea DES was added and vortexed	VA-DLLME	GC-μECD	0.01–0.08 μg/kg	63–119% (2–7%)	A hydrophilic DES was used in the sample pretreatment to reduce the matrix effect of olive oils.	[100]
Menthol:octanoic acid (1:4) (500 μL)	Diphenylamine	Apple, pear and orange (1000 mg)	Samples were homogenised	UA-DLLME	HPLC-FD	0.05 μg/L	96–108% (1–4%)	-	[101]
ChCl:butyric acid (1:2) (180 μL)	6 herbicides	Tea (5000 mg)	Samples were used without any pretreatment	HLLE-DLLME	GC-MS	0.0026–0.0084 μg/kg	70–89% (-)	NaCl was used as a separation agent. A hydrophilic DES (ChCl:phenol) was used as a disperser solvent. ACN was used as demulsifier agent.	[102]
**Non NADES**									
TBACl:2,3-butanediol (1:3) (500 μL)	Patulin	Apple, orange, peach, apricot, grape, kiwi, cherry and mango juices (3 mL)	Samples were diluted, extracted with ACN, centrifuged and the supernatant was mixed with PSA and MgSO_4_	UA-DLLME	UV-Vis	2.2 μg/L	90–107% (2–4%)	Acetone was used as emulsifier solvent.	[16]
N_4444_Cl:octanoic acid (1:2) (100 μL)	8 synthetic pigments	Carbonated drinks, tea beverage, fruit juices, and lactobacillus beverages (10 mL)	Carbonated drinks and tea beverage were used directly. Fuit juices and lactobacillus beverages were diluted 10 times and centrifuged to use the supernatant	VA-DLLME	HPLC-DAD	0.016–1.120 μg/L	75–103% (1–5%)	4 DESs were evaluated.	[86]
TOMAC:2-octanol (1:2) (800 μL)	3 sulfonamides	Apple, grape, peach and pear juices, and black tea (5 mL)	Samples were filtered and sealed	UA-DLLME	HPLC-UV	20–50 μg/L	81–104% (0–9%)	5 DESs were evaluated.	[87]
TBABr:malonic acid:hexanoic acid (1:1:1) (2000 mg)	2 sulfonamides	Chicken meat (1000 mg)	Samples were homogenized and liophilized	DLLME	HPLC-UV	3–7 μg/kg	86–109% (8%)	The DES decomposes when aqueous phase is injected, and the hexanoic acid is responsible for the extraction of the analytes.	[103]
BTEAB:eugenol (1:2) (75 mg)	3 sudan dyes	Chili sauce, chili powder and ketchup (8 mL)	Samples were mixed with MeOH, ultrasonicated, centrifuged and diluted	VA-DLLME	HPLC-DAD	0.5–1 μg/L	90–119% (0–7%)	0.5% NaCl (*w*/*v*) was added.	[104]
Menthol:dichloroacetic acid (1:2) (30 μL)	7 pesticides	Honey (5000 mg)	Samples were diluted with water, and acetone was used as an extraction solvent	DLLME	GC-FID	0.32–1.2 μg/kg	90–109% (1–8%)	Acetone was also used as a dispersive solvent. A cloudy state was formed after dispersion of the DES into the aqueous solution.	[105]
2-ethylhexyl 4-hydroxybenzoate:FWA52 (1:1) (-)	FWA52	Noodles, fish balls, mushroom and paper cups (1000 mg)	Samples were broken into powder	VA-DLLME	FD	0.045 μg/L	82–113% (5–10%)	FWA52 acts as an analyte and HBA, so the HDES was formed during the extraction of FWA52.	[106]
TOMAC: amylalcohol (4:1) (150 μL)	Folic acid	Wheat flour (5000 mg)	-	VA-DLLME	HPLC-UV	1.0 μg/kg	92–100% (2–6%)	3 DESs were prepared. The selected DES was mixed with MeOH.	[107]
N_8881_Br:decanoic acid (1:2) (200 μL)	6 fluoroquinolones	Milk, yogurt, honey, tap water and river water (5 mL)	(NH_4_)_2_SO_4_ was used to make the milk and yogurt demulsification and the honey extraction processes	SO-DLLME-BE	MECC-UV	6–10 μg/L	88–115% (1–7%)	HCl was added in the BE.	[108]
THACl:oleic acid (1:1) (200 μL)	Co	Biscuit, bitter chocolate wafers, white chocolate, corn, wheat, herbal tea, spinach, mint, tap, waste, river, and well water, chocolate milk, cow milk and red wine (5 mL)	All samples except water samples were mixed with H_2_O_2_:HNO_3_ (1:3, *v*/*v*) and a MWA digestion was performed	AA-DLLME	FAAS	0.04 μg/L	94–105% (2–4%)	CCD combined with RSM was used for optimization. 6 ionic HDESs were evaluated. The analytical method is based on the complex formation of Co(II) with dithizone.	[109]
BTEAC:thymol (1:2) (300 μL)	5 red dyes	Carbonated drink beverage, jelly and chocolate dragee (40 mL)	The carbonated drink was diluted and solid samples were dissolved	VA-DLLME	HPLC-UV	0.01–0.08 μg/L	94–101% (2–6%)	-	[110]
P_666(14)_Cl:pivalic acid (1:4) (200 μL)	Cd(II)	Waste, snow, rain, and tap water, cheese and milk (12 mL)	A wet digestion was applied with HNO_3_:H_2_O_2_ (3:1, *v*/*v*)	DLLME	FAAS	1.6 μg/L	95–99% (2–4%)	The BBD was used to determine the optimum conditions.	[111]
TOMAC:amyl alcohol (1:4) (100 μL)	2 pesticides	Pistachio (5000 mg)	Sample was milled and homogenized	QuEChERS-DLLME	HPLC-UV	1.5–3.0 μg/kg	96–99% (2–7%)	The QuEChERS step allowed a better extraction and clean-up.	[112]
TBACl:decanoic acid (1:3) (100 μL)	Ni(II)	Waste, sea, mineral and well water, onion, parsley and cigarette (30 mL)	-	UA-DLLME	FAAS	0.13 μg/L	97–105% (-)	THF was used as self-aggregation agent.	[113]
PChCl:dichloroacetic acid:dodecanoic acid (1:1:1) (55 μL)	4 antibiotics	Milk (7 mL)	ACN acted as proteins precipitation	SI-HLLE-DLLME	HPLC-DAD	2.0–2.8 μg/L	87–106% (5–6%)	AES tool was used for the assessment of the greenness of the proposed method.	[114]
Menthol:undecanol (1:2) (300 μL)	3 bisphenols	Canned fruits (500 mg)	Samples were homogenized and freeze-dried	DLLME-SFO	UHPLC-MS/MS	1.5–3.0 μg/kg	79–101% (1–5%)	ACN was used as dispersion solvent.	[115]
TOMAC:DL-lactic acid (1:3) (400 μL)	Cd and As	Sorghum wine (5 mL)	Dried sea snake, seahorse and petrel were added to sorghum wine and keep for 6 months	UA-DLLME	FAAS	0.08–0.30 μg/L	91–104% (3–8%)	8 DESs were compared. MeOH was used as dispersion solvent.	[116]
Aliquat 336:decanoic acid (1:2) (600 μL)	2 food dyes	Fruity pastel, smarties, ice cream, candy and jelly (8 mL)	Samples were dissolved in water, centrifuged and the supernatant was diluted	EA-DLLME	UV-Vis	2.0–2.9 μg/L	98–103% (-)	DES was mixed with acetic acid. CO_2_ was produced in an effervescent reaction (acetic acid and NaHCO_3_) and was used as the dispersive force for DES.	[117]
ZnCl_2_:acetamide (1:2) (350 μL)	V	Sea, waste, canal, mineral, tap and drinking water (25 mL). Apple, banana, tomato, spinach and cultivated mushroom (1000 mg)	-	The solutions were heated until become turbid and then were centrifuged	GFAAS	0.01 μg/kg 0.01 μg/L	96–100% (1–3%)	Triton X-114 was used to enhanced phase transfer ratio. A factorial design and CCD were applied in the optimization process.	[118]
FeCl_3_:phenol (1:5) (150 μL)	Pb(II)	Tap, lake and river water (10 mL), salted peanuts, chickpeas, roasted yellow corn, pistachios and almonds (2 mL)	Water samples were filtered. Food samples were digested with HNO_3_	VA-DLLME	FAAS	0.008 μg/L	92–101% (-)	α-benzoin oxime was used to enhance the ability of the DES to coordinate Pb(II).	[119]
ChCl:1-(o-tolyl)biguanide (1:1) (20 μL)	5 PFASs	Olive, sesame, sunflower, seed, corn, camellia seed, soybean, blended and vegetable oils (7000 mg)	Samples were homogenized and microextracted at 40 °C	Superparamagnetic nanofluid	UHPLC-MS	0.0003–0.0016 μg/kg	90–109% (5–8%)	The DES system based superparamagnetic nanofluid can retrieved by an external magnetic field without additional centrifugation.	[120]
TBABr:dodecanol (1:2) (1.5 μL)	67 terpenes	Cinnamon, cumin, fennel, clove, thyme and nutmeg (50 mg)	Spices were used as fine-grained powders, seeds and in small pieces	HS-SDME	GC-MS	141–25,920 μg/kg	- (-)	Extraction time and temperature significantly affect the extraction.	[121]
ChCl:butyric acid (1:2) (58 μL)	5 acidic pesticides	Tomato (50 mL)	Samples were cut, crushed, filtering the produced juice and diluted	SBME-DLLME-SFO	GC-MS	0.007–0.014 μg/L	86–99% (3–5%)	15% (*w*/*v*) of NaCl was added. A hydrophilic DES (ChCl:ethylene glicol) was used as elution/dispersive solvent.	[122]
TBACl:decanoic acid (3:1) (100 μL)	Pb(II)	Tobacco, onion and parsley (30 mL)	Samples were digested with HNO_3_ and filtered	UA-DLLME	FAAS	4.4 μg/L	94–105% (4%)	THF was used as an emulsifier agent. PBD was used to determine the optimum conditions.	[123]
P_666(14)_Cl:tetradecyl alcohol (1:3) (10 mg)	5 benzoylurea pesticides	Green tea, oolong tea, grapefruit water and lemon water (8 mL)	Samples were filtered	UA-DLLME	HPLC-UV	0.30–0.60 μg/L	77–101% (0–7%)	-	[124]
TBABr:decanoic acid (1:3) (100 μL)	4 neonicotinoid insecticides	Water, soil and egg yolk (10 mL)	Water samples were filtered. Soil samples were air-dried, ground, sifted and extracted with anhydrous Na_2_SO_4_ and anhydrous NaAc. Egg samples were mixed with anhydrous Na_2_SO_4_ and 1% (*v*/*v*) acetic acid in ACN	DLLME	HPLC-UV	1–3 μg/L	60–114% (<10%)	ACN and SDS were used as disperser solvents.	[125]
ChCl:decanoic acid (1:2) (63 μL)	7 pesticides	Milk (5 mL)	-	DLLME-SFO	GC-FID	0.9–3.9 μg/L	64–89% (3–6%)	ChCl:ethylene glycol was used as extraction/disperser solvent.	[126]
Dichloroacetic acid:L-menthol:n-butanol (4:1:1) (100 μL)	10 pesticides	Green tea, and rose water, lemon balm, mint, and pussy willow distillates (50 mL)	The green tea was added to boiling water, centrifuged and filtrated	DLLME	GC-FID	0.11–0.23 μg/L	86–112% (1–7%)	MeOH was used as disperser solvent.	[127]
Dichloroacetic acid:butanol:menthol (2:1:1) (85 μL)	10 pesticides	Tomato (10,000 mg)	Samples were chopped, squeezed and homogenized	MWA-DLLME	GC-FID	0.42–0.74 μg/kg	85–103% (-)	ACN was used as a dispersive solvent.	[128]

μECD: micro electron capture detector; AA: air-assisted; ACN: acetonitrile; AES: Analytical Eco-Scale; BBD: Box-Behnken design; BDP-d_4_: dibutyl phthalate-3,4,5,6-d_4_; BE: back extraction; BTEAB: benzyltriethylammonium bromide; BTEAC: benzyltriethylammonium chloride; CCD: central composite design; ChCl: choline chloride; DAD: diode-array detector; DEHA: bis(2-ethylhexyl) adipate; DES: deep eutectic solvent; DHP: dihexyl phthalate; DHP-d_4_: dihexyl phthalate-3,4,5,6-d_4_; DLLME: dispersive liquid–liquid microextraction; DNOP: di-n-octyl phthalate; EA: effervescence assisted; EU: European Union; FAAS: flame atomic absorption spectrometry; FD: fluorescence detector; FID: flame ionization detector; FWA52: fluorescent brightener 52; GC: gas chromatography; GFAAS: graphite furnace atomic absorption spectrophotometer; HBA: hydrogen bond acceptor; HDES: hydrophobic deep eutectic solvent; HLLE: homogenous liquid–liquid extraction; HPLC: high-performance liquid chromatography; HS-SDME: headspace single-drop microextraction; IS: internal standard; LOD: limit of detection; MECC: micellar electrokinetic capillary chromatography; MeOH: methanol; mNP: magnetic nanoparticle; MS/MS: tandem mass spectrometry; MS: mass spectrometry; MWA: microwave-assisted; N_8881_Br: methyltrioctyl ammonium bromide; NaAc: sodium acetate; NADES: natural deep eutectic solvent; P_666(14)_Cl: trihexyltetradecylphosphonium chloride; PAE: phthalic acid ester; PAH: polycyclic aromatic hydrocarbon; PBD: Plackett–Burman design; PChCl: phosphocholine chloride; PFAS: perfluoroalkyl substance; PSA: primary secondary amine; QuEChERS: quick, easy, cheap, effective, rugged and safe; RSD: relative standard deviation; RSM: response surface methodology; SBME: stir bar sorptive extraction; SDS: sodium dodecyl sulfate; SFO: solidification of the floating organic drop; SI-HLLE: salt induced-homogenous liquid–liquid extraction; SLE: solid–liquid extraction; SO: salting out-assisted; TBABr: tetrabutylammonium bromide; TBACl: tetrabutylammonium chloride; THACl: tetraheptylammonium chloride; THF: tetrahydrofuran; TOMAC: trioctylmethylammonium chloride; UA: ultrasound-assisted; UHPLC: ultra-high-performance liquid chromatography; UV: ultraviolet; VA: vortex-assisted; Vis: visible; VWD: variable wavelength detector.
